# A Cost-Effective Microfluidic Device to Teach the Principles of Electrophoresis and Electroosmosis

**DOI:** 10.1021/acs.jchemed.2c01028

**Published:** 2023-06-20

**Authors:** Tyler A. Shaffer, Carlos U. Herrada, Avery M. Walker, Laura D. Casto-Boggess, Lisa A. Holland, Timothy R. Johnson, Megan E. Jones, Yousef S. Elshamy

**Affiliations:** † C. Eugene Bennett Department of Chemistry, West Virginia University, Morgantown, West Virginia 26505, United States; ‡ Department of Chemistry, St. Norbert College, De Pere, Wisconsin 54115, United States

**Keywords:** Upper-Division Undergraduate, Laboratory Instruction, Hands-On Learning/Manipulatives

## Abstract

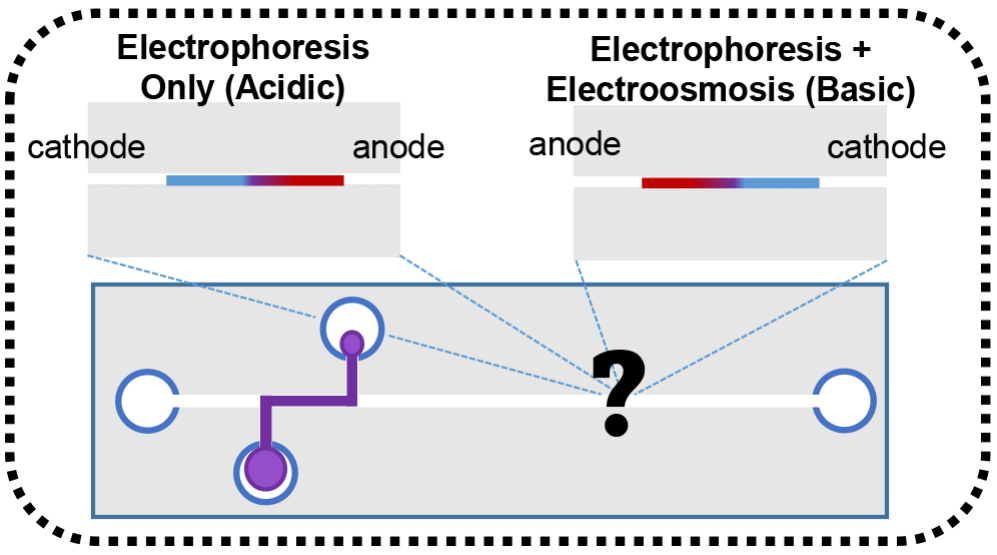

Electrophoresis is integral to analytical and biochemistry experiences in undergraduate education; however, fundamental principles of the method are often taught in upper-level laboratories through hands-on experiences. A laboratory activity is reported that teaches the concepts of electrophoretic mobility and electroosmotic flow. A single reuseable instrument, called a mini-E, costs 37 USD and consists of a DC power supply, a voltmeter, platinum electrodes, and a chip cast in polydimethylsiloxane. This activity uses common reagents costing only 0.02 USD per student. Experiments are devised that allow students to investigate the properties of electrophoretic flow and electroosmotic flow by separating the two commonly used food dyeing agents Brilliant Blue FCF and Allura Red AC in vinegar and in a solution of ammonium hydroxide. A dark-purple mixture of these dyes is separated into red and blue bands that are easily visualized. The migration order of the dyes differs when the separation is performed under conditions of reversed polarity and suppressed electroosmotic flow (vinegar) compared to conditions of normal polarity and active electroosmotic flow (ammonium hydroxide). When delivered to chemistry majors, students had a significant gain in their ability to apply the concepts of electroosmosis and electrophoresis to predict analyte migration. Although this activity targets upper-level chemistry content, it can also be adapted for other laboratory experiences.

## Introduction

Electrophoresis is foundational to biomolecular separations, and in the modern form adapted to fused silica capillary,^1^ capillary electrophoresis has become a key method for bioanalysis.^2^ Capillary electrophoresis has been integral for the first whole genome shotgun sequencing of the human genome,^3^ for human forensic identification,^4^ and for biological therapeutics.^5^ As a result of the significance of electrophoresis, the method is identified in the analytical and biochemistry curricula for a chemistry degree program certified by the American Chemistry Society.^6^ A recent survey also confirms that electrophoresis is prevalent in the biochemistry teaching laboratory.^7^ Indeed, electrophoresis experiments are described for upper-level courses in analytical chemistry^8,9^ and biochemistry^10^ laboratories. Efforts to enhance the role of laboratory instruction in chemical education have drawn new attention.^11^ The activity described in this report allows upper-level students to learn the key concepts of this analytical technique. A key innovation of the miniaturized electrophoresis system (mini-E) is that participants visualize the separation occurring before them and learn capillary electrophoresis concepts in a personalized way. The consumable reagents cost 0.02 USD, and the mini-E costs 37 USD. The mini-E separation can be delivered as a traditional laboratory, for example, in a section of 24 students who are supervised by a teaching assistant or the chemistry instructor.

A laboratory experiment was designed with four learning objectives which fall into two of the learning levels described by Bloom’s taxonomy as understanding and application.^12^ The three learning objectives for this experiment were based on understanding electrophoresis transport mechanisms of electrophoretic mobility, electroosmosis, and the description of these mobilities with vectors. The principles taught through knowledge and understanding rely on a simple memorization of facts and principles to think through simple problems. These facts and principles were introduced through the prelaboratory lecture and through the experimental handout itself and not from the hands-on experiment with the microfluidic device. A fourth learning objective for this experiment centered on the application of transport mechanisms to predict migration order, which was then compared to the experimentally observed migration order of the red and blue zones of dye. The application-based learning involved more complex combinations of electroosmotic and electrophoretic transport through vector analysis. This process benefited from the visual nature of the experiment, enforcing a deeper learning of the concept beyond memorization.

## Background

**Figure 1:**
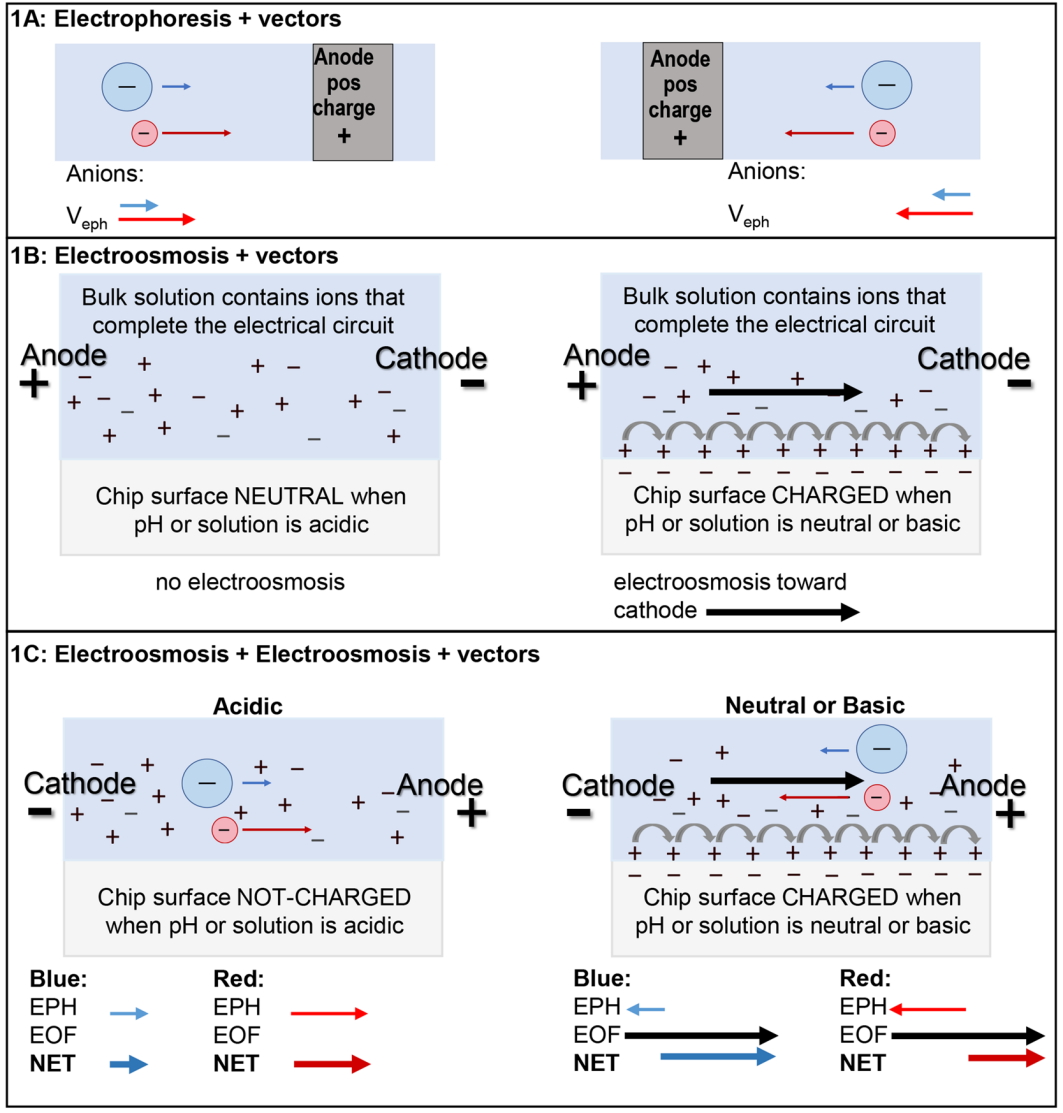
(A) Diagrams showing ion movement based only on electrophoretic mobility (EPH). The smaller red anion moves to the anode faster than the larger blue anion. (B) Illustration of the absence or presence of the bulk electroosmotic flow (EOF). The vector below the image shows the direction and magnitude of bulk flow in the capillary. (C) Analysis of EPH and EOF. Under acidic conditions and reversed polarity, the EOF is absent. Under basic conditions and normal polarity, the EOF and EPH are combined by using vectors to show how the direction and magnitude of each transport mechanism contributes to the net motion.

The foundational understanding of the principles of electrophoretic mobility and electroosmosis were described in the laboratory introduction and prelaboratory questions. The concepts outlined in the laboratory handout described the electrophoretic movement in the capillary due to charge attraction as well as differences in ion velocity caused by frictional drag. The effect of the charge-to-size ratio on electrophoretic mobility of an anion and the vectors associated with this transport toward the anode are depicted in Figure 1A. The length of the vector is equal to the magnitude of the transport, with a longer vector indicating greater velocity. Additionally, the position of the anode determines the direction of the transport. The presence or absence of an electroosmotic flow is fundamental to capillary electrophoresis. The polydimethylsiloxane material used to fabricate the device contains charged silica functional groups,^13−15^ which create a significant negative charge when the surface is in equilibrium with an aqueous solution at a pH above 6.4.^13,14^ Under these conditions of neutral or basic pH, the negatively charged surface attracts positive counterions in the solution. In the presence of an electric field, these cations move toward the cathode in a trainlike fashion along the capillary surface, as depicted in the simplified concept diagram of electroosmosis shown in Figure 1B. For a more detailed explanation of electroosmotic flow and depiction, see Figure S1 in the upper-division student handout. This movement, depicted as an additional vector, causes a bulk flow to occur toward the cathode.

The electroosmotic flow within the capillary is affected by the pH of the aqueous solution in the channel. Under acidic conditions there is no significant electroosmotic flow present. In a separation without electroosmotic flow, no vector is assigned to this mode of transport, and the analyte migration is determined solely by electrophoretic mobility, as shown in Figure 1C for a separation under acidic conditions. The analytes with the same charge but greater size travel slower than the smaller analytes. However, for a separation under neutral or basic conditions, as shown in Figure 1C, the surface of the capillary will be charged, and electroosmotic flow will be present. The electrophoretic movement is in the opposite direction of the electroosmotic flow. The use of vectors reinforces the fact that the migration order of the analytes is opposite of that observed in the absence of electroosmotic flow.

**Figure 2:**
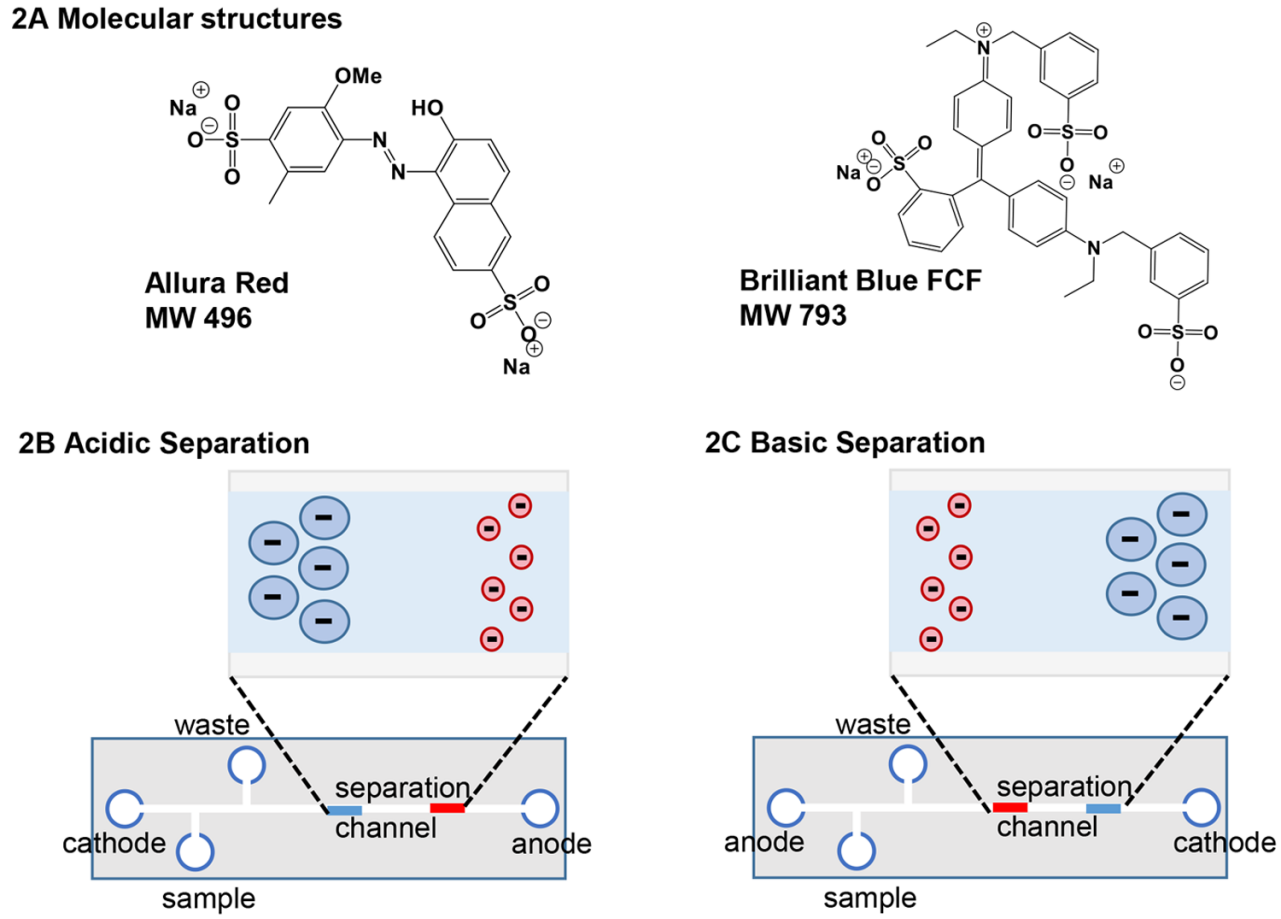
(A) Dye structures. (B) Separation of the dyes under acidic conditions. (C) Separation of the dyes under basic conditions.

The elution order for this experiment is evaluated by the students with knowledge of the structures of the dyes. As shown in Figure 2A, Allura Red AC has a lower mass than Brilliant Blue FCF; however, both analytes are anionic with a net charge of 2–. As depicted in Figure 2B, when an acidic separation solution is used in a microfluidic chip or in a capillary electrophoresis instrument, both dyes migrate toward the anodic reservoir, and the Allura Red AC migrates faster than Brilliant Blue FCF. When a basic solution is used, however, the electroosmotic flow will overcome electrophoretic forces in the capillary, and both dyes migrate toward the cathodic reservoir. In this case the net mobility of Brilliant Blue FCF is greater than that of Allura Red AC, and Brilliant Blue FCF will elute first (Figure 2C).

## Hazards and Safety

The use of ammonium hydroxide in the second portion of the experiment requires standard laboratory personal protective equipment. When combined, the DC power converters deliver a direct current of 0.5 A at 96 V. As designed, the system current is limited by a 500 kΩ resistor and produces a current of 192 μA. With dry skin, the hazard of potential electrical discharge is not a risk, and contact with the electrical system will produce no noticeable effect to the user. With wet skin, contact with both electrodes may cause a tingling sensation.^16^ This sensation can be avoided by using nitrile gloves or by direct handling of the electrodes with dry skin. The use of any food-grade materials in the laboratory requires the same degree of caution applied when used outside of the laboratory (e.g., at home).

## Experiments

The experimental protocol leads the students to visualize the migration order of the Allura Red AC and Brilliant Blue FCF dyes in the microfluidic channel using the acidic (i.e., filtered vinegar, which is 0.8 M acetic acid) and then basic (0.1 M ammonium hydroxide) separation solutions. For these experiments, the dye standards should be prepared in the background electrolyte (i.e., either 0.8 M acetic acid or 0.1 M ammonium hydroxide) to a concentration of 60 mM Allura Red AC and 40 mM Brilliant Blue FCF. The students use a microfluidic device to perform electrophoresis first with suppressed electroosmotic flow and reversed polarity to visualize the red band migrating faster than the blue band toward the end of the separation channel. Next, the students use a microfluidic device to perform electrophoresis with significant electroosmotic flow and normal polarity to visualize the blue band migrating faster than the red band toward the end of the separation channel. These experiments provide a visual cue and opportunity for reflection about the transport mechanisms that lead to this reversal in the order of dye migration.

**Figure 3:**
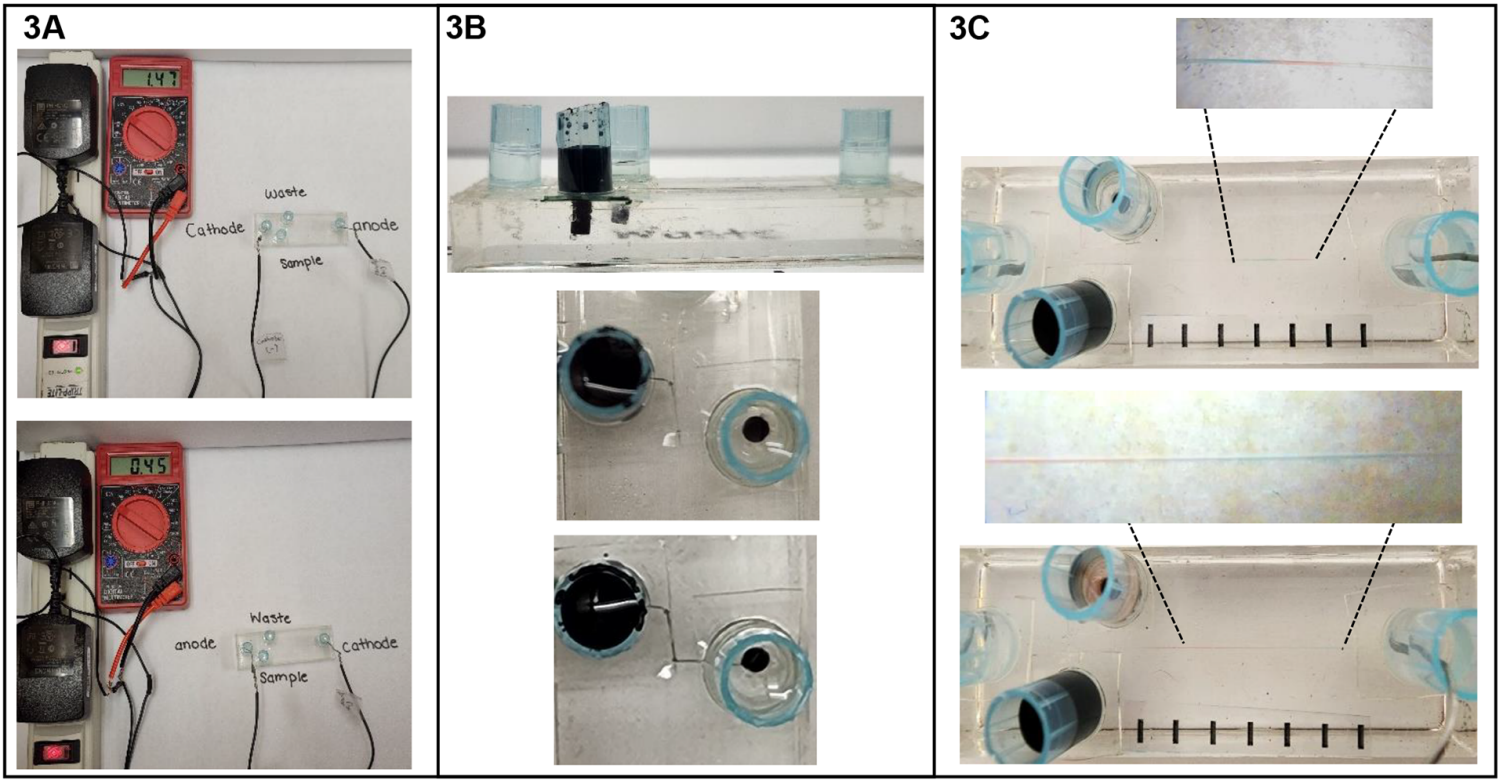
(A) Image of the setup used to perform these separations, including the twin 48 V DC converters in series, the 500 kΩ resistor/multimeter readout setup, and the polydimethylsiloxane chip. (B) Images of the chip depicting the geometry of the separation and injection channels created by using the fishing line in the mold to define the channels. Additionally, the method of injection is shown. The sample well in the upper image contains 300 μL of the dye solution. The lower images show dye movement through the injection zone due to intentional siphoning. (C) Images of the separation of the dye mixture with the (top) acidic and (bottom) basic separation conditions.

The instrumental setup that drives the separation is shown in Figure 3A and costs 37 USD. The cost of the polydimethylsiloxane used for the microfluidic device is only 4.27 USD, whereas the cost of the other parts (DC inverter power supply, power supply clips, resistor, electrodes, multimeter) is 31.00 USD (see Table S1 in the instructor handout). The multimeter is useful to determine whether air bubbles have been inadvertently introduced into the channels. In principle, the experiment can be conducted without the aid of the multimeter. The electrodes are constructed from platinum-plated earrings, as platinum is generally considered resistant to oxidation, for example in aqueous electrolysis, and because it is the material of choice in commercial capillary electrophoresis instruments. Although other materials were not used in the development of this laboratory experiment, electrodes constructed from other conductive materials may be acceptable if oxidation is not a concern. The process of constructing the electrodes from platinum-plated earring hooks used for jewelry crafting (for the vendor and part number, see Table S1 in the instructor handout) involves straightening the earring so that it is easily be placed in the reagent wells of the device. The wire connected to the power supply is wrapped around the back end of the earring, as shown in Figure S5C in the instructor handout. The power supply comprises two 48 V DC converters, driving the electrophoresis with an applied voltage of 96 V. Laboratory-grade electrophoresis power supplies can be used, provided that the operator can apply a voltage of approximately 100 V. If a higher voltage is applied, the user should ensure that the separation current is low (e.g., ≤10 μA) to prevent Joule heating.

The microfluidic chip shown in Figure 3B,C is constructed as a single channel comprising a 0.5 cm double-T injection zone in line with a 4 cm separation zone. Although polydimethylsiloxane devices are typically cast as open channels that are sealed on a glass surface, the separation channel in Figure 3 is internally cast in polydimethylsiloxane as outlined in the instructor handout. This process involves floating fishing line within the mold using inexpensive magnets, adding the polydimethylsiloxane, and allowing it to cure. Once it is cured, the magnets and fishing line are easily removed. The device is fabricated as outlined in the instructor handout using polydimethylsiloxane. Other materials can be used to construct the device, provided that they sustain an electroosmotic flow. Additionally, the instructor may choose to fabricate a device of different channel dimensions. The channel dimension of 150 μm was selected for this laboratory experiment because smaller dimensions may be easier to plug with particulate while larger dimensions generate higher separation currents, leading to Joule heating. This use of a solid polydimethylsiloxane device was found to be more robust when handled by the students. The polydimethylsiloxane device can be used repeatedly and stored indefinitely. At the time of this report, a single device has been used approximately 20 times.

The process of introducing the dye mixture across the double-T injection zone (Figure 3B) is facilitated by siphoning the sample from the sample reservoir to the waste reservoir. This is achieved by removing a 100 μL volume of the background electrolyte from the waste reservoir while leaving the other three reservoirs filled to a volume of 300 μL. Once the dye mixture has filled the double-T injection zone, the liquid is replaced in the waste reservoir to prevent any further siphoning, and the separation voltage is applied. The separation is complete within 3 min with the background electrolyte composed of vinegar, which is 0.8 M acetic acid (Figure 3C), and within 5 min with the background electrolyte composed of 0.1 M ammonium hydroxide. The blue and red bands are visualized as shown in the upper (vinegar) and lower (ammonium hydroxide) images in Figure 3C. When the experiment is delivered to upper-level chemistry students, after experimental procedures are communicated to the students, they proceed with the sample injection and separation. In a 2 hour laboratory period, the students have adequate time to repeat the experiment if required. The mini-E experiment may be adapted to introductory level chemistry students (see the introductory student handout). In this case, after the protocol is communicated, it is also recommended that the experiment be demonstrated by the instructor. This requires the instructor to complete the chip setup (Figure 3A) as well as the sample loading (Figure 3B). The students then observe the separation (Figure 3C) and with the remaining time are given the opportunity to repeat the dye injection and complete the separations independently.

## Assessment of Experiments

Questions were developed to accompany the laboratory exercise in order to assess the learning outcomes. The activity was evaluated by administering a 12 question assessment to 10 upper-level chemistry majors. The process involved a presentation on basic principles of capillary electrophoresis followed by the laboratory experience. The students viewed a brief (∼10 minute) lecture presentation on electrophoresis. The students were asked to complete the assessment following the lecture but before reading the laboratory activity. Upon completing the laboratory activity, the students immediately completed the assessment again. The questions were designed to address the concepts of electrophoretic mobility, electroosmotic flow, vector analysis, and migration order predictions at lower-level learning (i.e., seven questions related to knowledge and understanding) as well as higher-level learning (i.e., five questions related to application). The students had a statistically significant improvement in all categories as calculated using a Student’s *t* test. For the seven questions related to knowledge and understanding, the number of correct responses given increased from 5._2_ ± 1._4_ to 6._8_ ± 0.4_2_ before and after the activity, respectively. This improvement in scores is statistically significant (ρ = 0.05). These questions focused on electrophoretic mobility, and learning can be attributed not only to the experiment but also to the handout and prelaboratory presentation. A more substantial improvement was observed from the application-based questions. The scores for the number of correct answers for a total of five questions asked increased from 2._3_ ± 1._2_ to 4.2_0_ ± 0.9_2_ for the assessment delivered before and after the laboratory experiment, respectively. These questions show a statistically significant gain (ρ = 0.05). The larger improvement in the applications portion of the assessment was attributed to the visual and kinesthetic nature of the microfluidic separation, allowing the participants to see the concepts of electrophoresis and electroosmosis in real-time.

## Limitations

Some considerations are noted for the experiment. If the separation channel is inadvertently loaded with an air bubble or excess dye, then the instructor must be prepared to flush and refill the device. The polydimethylsiloxane may become discolored if the food coloring is used at concentrations higher than 60 mM for Allura Red and 40 mM for Brilliant Blue FCF. If the device becomes stained, any discoloration can be removed by rinsing with water or isopropyl alcohol. When the channel is filled with the 0.1 M ammonium hydroxide, occasionally the device will appear hazy. This change in appearance, due to the gas permeability of polydimethylsiloxane^17^ and the evolution of ammonia gas, is corrected by heating the device to 40 °C overnight. The tape that is used to seal the reservoirs to the polydimethylsiloxane device requires moderate manual dexterity when applied to the device. Solvents can delaminate the tape from the polydimethylsiloxane surface; however, if the surface and tape are allowed to dry, it can be reapplied. During the separation, the dye bands will diffuse more with increasing separation time. This band broadening is less pronounced in vinegar, as these runs are significantly faster than those obtained with ammonium hydroxide. Finally, if the channel is cast with fishing line designed to be invisible in water, it will be difficult to check that the fishing line remains connected to the channels in the uncured polydimethylsiloxane. Therefore, the polydimethylsiloxane should not be disturbed until it is cured.

## Conclusions and Future Directions

The activity outlined in this report is a cost-effective strategy to teach concepts that are fundamental to capillary electrophoresis. The experiment has been assessed in upper-level chemistry instruction but can be adapted to lower-level chemistry instruction as well. Aside from the 0.1 M ammonium hydroxide, all reagents used in this experiment can be sourced from household products. By actively viewing the separation of the blue and red food dye the participants gain a deeper understanding of the underlying principles and apply their understanding of electrophoretic mobility and electroosmosis. This empowers the students to predict migration order in electrophoresis systems. Ultimately, the device can be delivered as a prelaboratory exercise prior to exposure to a commercial capillary electrophoresis system, for which student contact time may be limited. When delivered to upper-level chemistry majors, the students gain an appreciation of the utility of electrophoresis in society in forensics and analyses of biological pharmaceuticals. Future directions for this research include changing the casting process, for example, by increasing the length of the channel or using 3D printing techniques to create devices with polymeric materials that sustain electroosmotic flow.^18^ Additionally, the experiment will be evaluated for the effects of other aqueous electrolyte solutions and analytes.

## Supporting Information Available

Lecture introducing the students to electrophoresis (MP4)
Video showing ammonium hydroxide separation (MP4)
Video showing vinegar separation (MP4)
Video showing chip casting (MP4)
Instructor handout (PDF, DOCX)
Upper-division student handout (PDF, DOCX)
Introductory student handout (PDF, DOCX)
Credit statement (PDF, DOCX)
