# Supplementary material for: A Cost-Effective Microfluidic Device to Teach the Principles of Electrophoresis and Electroosmosis
Source: J Chem Educ. 2023 Jun 20;100(7):2782–8. doi: 10.1021/acs.jchemed.2c01028 (PMC10339723; doi:10.1021/acs.jchemed.2c01028)
Supplement: Supplementary file 6 — ed2c01028_si_006.docx [file ed2c01028_si_006.docx]

**A Cost-Effective Microfluidic Device to Teach the Principles of Electrophoresis and Electroosmosis**

Tyler A. Shaffer^1^, Carlos U. Herrada^2^, Avery M. Walker,^1^ Laura D. Casto-Boggess^1^, Lisa A. Holland^1^*, Timothy R. Johnson^1^, Megan E. Jones,^1^ Yousef S. Elshamy^1^

^1^C. Eugene Bennett Department of Chemistry, West Virginia University, Morgantown, WV 26505, United States of America

^2^Department of Chemistry, St. Norbert College, De Pere, WI 54115, United States of America

*Corresponding Author, Lisa.Holland@mail.wvu.edu

**ABSTRACT**

This material includes the Instructor handout. Protocols are described to manufacture chips, dye solutions, and electrical setups.

**TABLE OF CONTENTS**

**Information Page**

**Laboratory Protocol**

Materials S-1

Casting protocol S-2

Preparing aqueous dye solutions S-5

Preparing electrical setups S-5

Making wells S-8

Attaching wells S-9

Preparing for laboratory (filling the chips) S-10

Clean up S-11

**Parts and Prices**

Cost summary S-12

**Instructor Guide to Laboratory Preparation**

**Materials needed for 1 student**

1 PDMS microfluidic chip

1 electrical setup

10 mL of filtered vinegar filtered background electrolyte

10 mL of 0.1M ammonium hydroxide background electrolyte (AMOH)

3 mL disposable syringe with 3mm O.D. tubing

0.3 mL of Dye mix (vinegar)

0.3 mL of Dye mix (AMOH)

Optional:

1 flashlight (to better see dye color in channel)

1 Fresnel Lens/magnifying glass (to better see dye color in channel)

Stadia lines (Gradients may be added to the chip via a printable transparency)

**Important Notes**

- It is pertinent that one is careful when setting the chips up for use, exertion of too much force on the upper portion of the wells may dislodge the VHB tape from the chip
- If dye solution made in advance be sure to shake well or vortex to ensure all dye is dissolved into solution
  - Be sure to vortex and centrifuge dye master stocks, as they are made very close to solubility limit, to make sure no solid dye particles make it into the capillary
- Commercial food dyes may be used in a 2:1 red to blue ratio but these commercial dyes have the propensity to stain the PDMS chips, especially in the sample and waste well areas (For this reason, dye standards for the most part were used)
  - Staining may be remediated by rinsing the chips with water or isopropanol.

**Casting PDMS Devices**

1. A casting jig is made (**Figure S1**). This jig used standard microscope slides as sidewalls a Lexan piece approximately double the thickness of a microscope slide with holes drilled halfway through in order to index the 3 mm magnets used to form the bottom well portions.

**
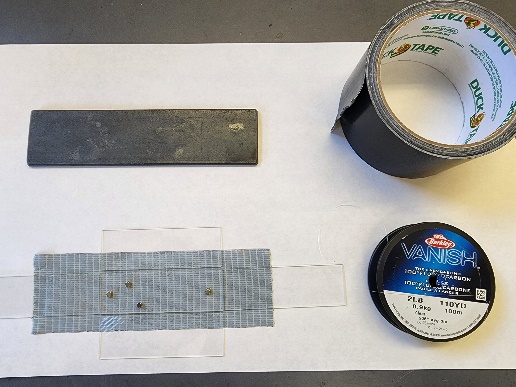
**

**Figure S1.** Equipment used for casting including the casting jig (bottom left) steel bar (top left), 150-micron monofilament fishing line (bottom right) and duct tape (top right).

2. The jig is wrapped in duct tape to seal and placed over a 6-inch piece of 1/8 inch thick steel.

3. 150-micron monofilament fishing line is tied in the double channel design and sandwiched at the ends between two 3mm magnets (**Figure S2**).

**
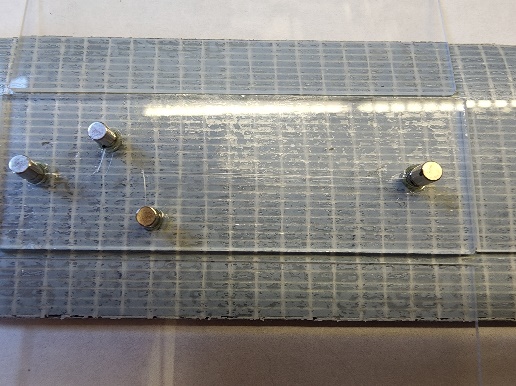
**

**Figure S2.** Casting jig with 150 micron monofilament fishing line to mold channels.

4. Jig is folded up (**Figure S3**) and wrapped in tape

**
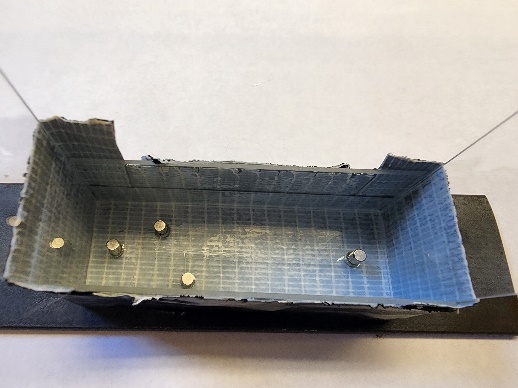
**

**Figure S3.** Folded jig ready to receive PDMS

5. PDMS is mixed in a 10:1 PDMS to Activator ratio.

6. Approximately 19g of PDMS is used to cast 1 Chip

7. PDMS is left to cure in a 65°C oven for 2 hours or overnight at 45°C

8. Chips were released from the jig by carefully inserting a razor blade between the microscope slides and the PDMS until the slides could be removed without tearing the PDMS

**Preparing the Dye solutions**

1. Dye solutions are first prepared in the form of master stocks, 400 mM allura red AC and 200 mM brilliant blue FCF.

2. These master stocks were then diluted by adding 40 µL of the Brilliant blue stock and 120 µL of allura red AC stock to a 1.5 mL PCR vial and filling with background electrolyte.

3. If using commercial dyes dilute 4 drops of red food coloring and 2 drops of blue food coloring in 5mL of background electrolyte.

**Preparing the Electrical Setups**

1. To make the electrical setups (**Figure S4**) used in the experiment, take 2 of the 48V invertors and snip the male adapter off the end of each.

2. Peel the two wires apart from each other on both invertors.


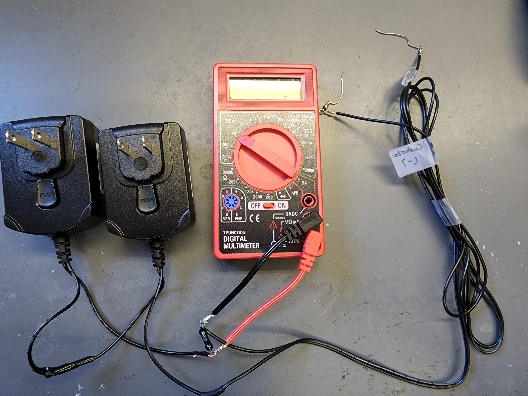
3. Locate the negative wire of one invertor and the positive wire of the other (positive wire is denoted with dashed grey line) and cut each at 3 inches or 7.5 cm long and solder them together (**Figure S5A**).

**Figure S4:** Finished electrical setup with 96V power source, multimeter readout, and earring electrodes.


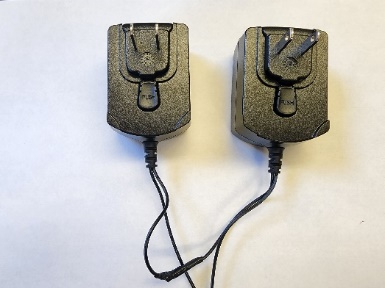


**Figure S5A:** This is a reference image depicting twin 48V DC invertors wired in series

4. Take the leads that came with your multimeter and cut them at 3 inches or 7.5 cm long as well (want the “banana” type ends) (**Figure S5B**).

5. Cut the remaining negative wire about halfway down.

6. Attach red lead, with solder, to 500 kΩ Resistor and piece of negative wire leading to invertor (**Figure S5B**).

7. Attach black lead along with rest of negative wire cut to opposite endo f resistor (with solder) (**Figure S5B**).

8. The wires connected to the electrodes are labelled as anode and cathode, respectively. Note that the anode wires are marked by the manufacturer with a dashed line down the length of the wire.


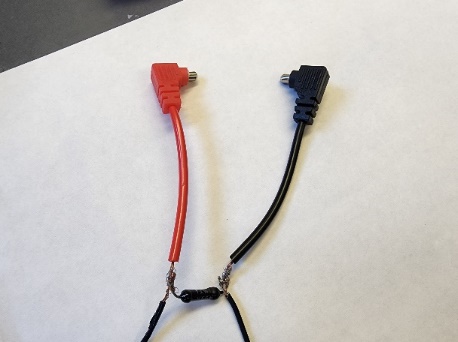


**Figure S5B:** This reference image depicts the modified multimeter lead wired together with the 500 kΩ resistor


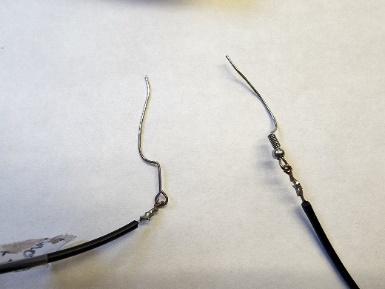
9. Strip ends of each wire and wrap wire around the earing attachment loop of an earring hook and solder in place (**Figure S5C**).

**Figure S5C:** Image depicting the attachment of the earing hook electrodes to the electrical setup

**Making Wells:**

1. Start with a micropipette tip and microscope slide cleaned with isopropanol.

2. With a lighter, melt the end of the tip as shown in (Figure S6)

3. Quickly tamp tip into microscope slide to create a flat surface (Figure S6A) making sure to keep tip as straight as possible.

4. Cut pipette tip above second ring (approximately 1.3 cm) above the newly made flat surface of tip as shown in (Figure S6B-D)


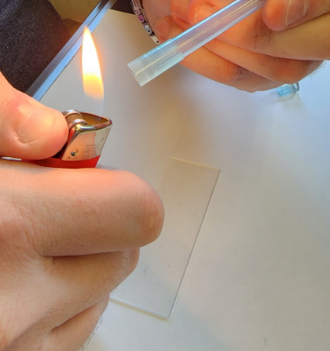

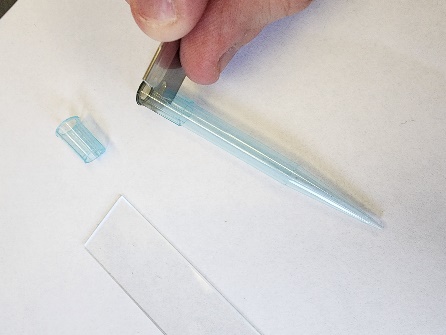


**A**

**C**


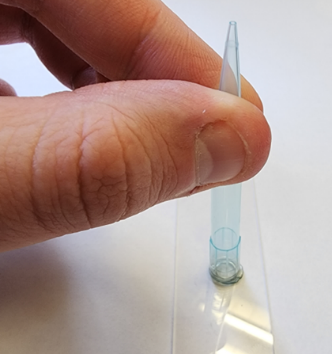

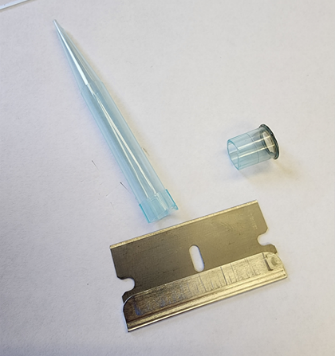


**D**

**B**

**Figure S6:** (A) Image depicting the melting of pipette tip end using a lighter. (B) the heated tip is quickly pressed on a clean glass slide to create a flat surface. (C) the pipet tip is trimmed with a razor blade (D) to size.

**Attaching Wells:**

1. Roll out and cut approximately 1.1 Cm of VHB tape onto microscope slide (Figure S7A-B)

2. Using a 6mm biopsy punch cut a hole in the center of the VHB tape segment cut in the previous step (Figure S7C)

3. Peel off tape, remove protective backing and attach to chip with the cast well in the center of the cut hole in the tape (Figure S7D-E)

4. Place tip well on top of tape (Figure S7F)

5. Repeat for all four wells and let adhesive cure in 45°C oven for 1 hour or 72 hours at room temperature for full bond strength

**
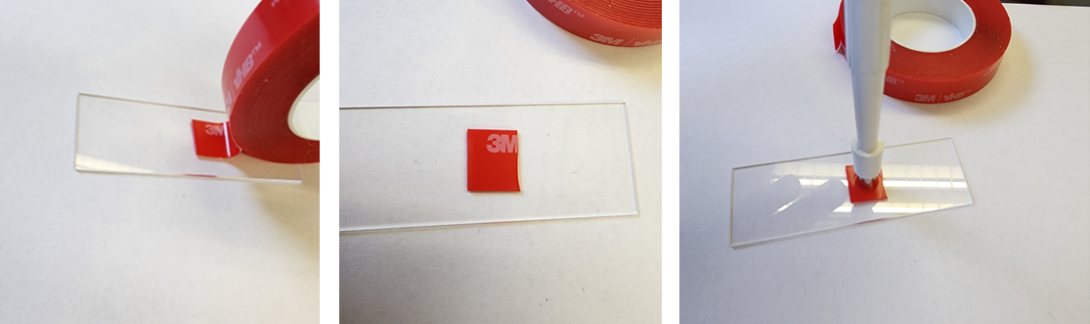
**

**C**

**B**

**A**

**F**

**D**

**E**


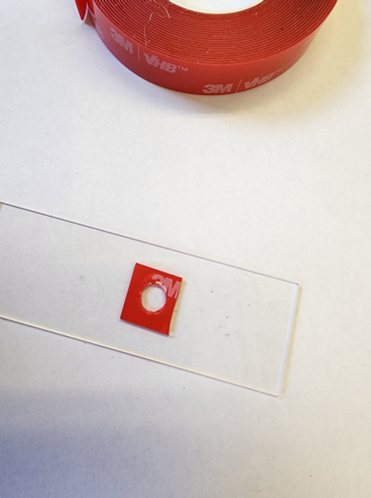

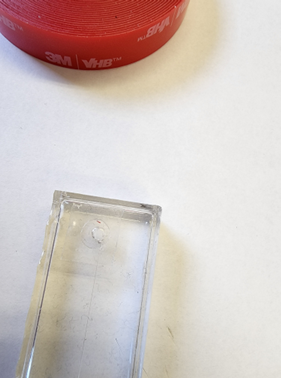

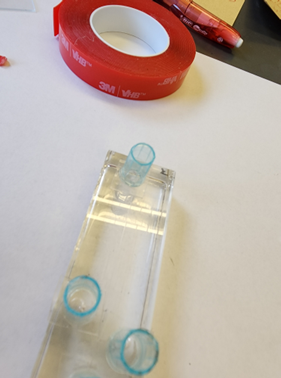


**Figure S7** Images depict the cutting of a section of VHB tape to attached the wells to the chip. (A) The tape is placed directly on a glass slide (B) and trimmed to size. (C) A biopsy punch is then used to create a hole in the tape. (D) The punched tape is removed leaving a hole. (E) The protective red layer is removed with forceps and the tape is place on the chip over the port. (F) With the tape in place the wells are pressed onto the tape.

**Preparing for Laboratory (Filling the chips)**

1. For one student to complete the first experiment, 10 mL of filtered vinegar, 10 mL filtered 0.1M ammonium hydroxide (AMOH), 0.3 mL of dye mix (vinegar), and 0.3 mL of dye mix (AMOH) are needed. Filtering of solutions serves to keep particulate out of the 150-micron capillary of the PDMS chip

2. Insert syringe tip (Figure S8) into the bottom of each well (Figure S9) and gently push solution into capillary. It may be necessary to make secondary passes to wells to fully remove bubbles. If bubbles are stubborn a good method to remove them is to fill the well halfway, insert the syringe, and suck fluid up to backfill channel.

3. After filling the channel, proceed to remove all fluid from the top portion of all wells

4. Fill all wells besides sample well with 300 µL of vinegar/AMOH solution and fill the sample well with 300 µL of dye mix. Insert electrodes (unplugged, or switched off if using a surge protector)

5. Remove 200 µL of background electrolyte from the waste well and allow dye to siphon into bottom of well until small purple-black dot appears

6. Transfer 200 µL of background electrolyte back into waste well and turn on/plug in the electrics


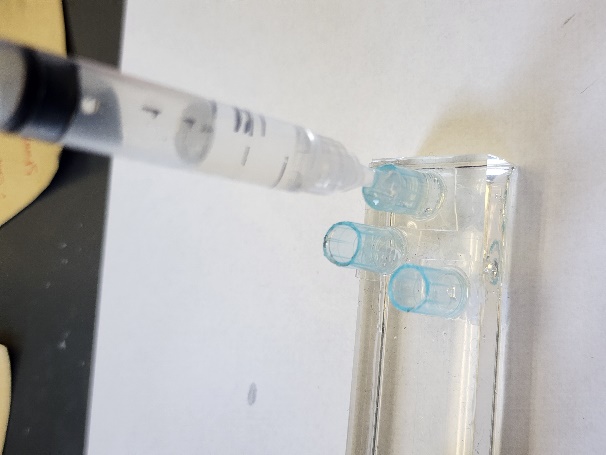

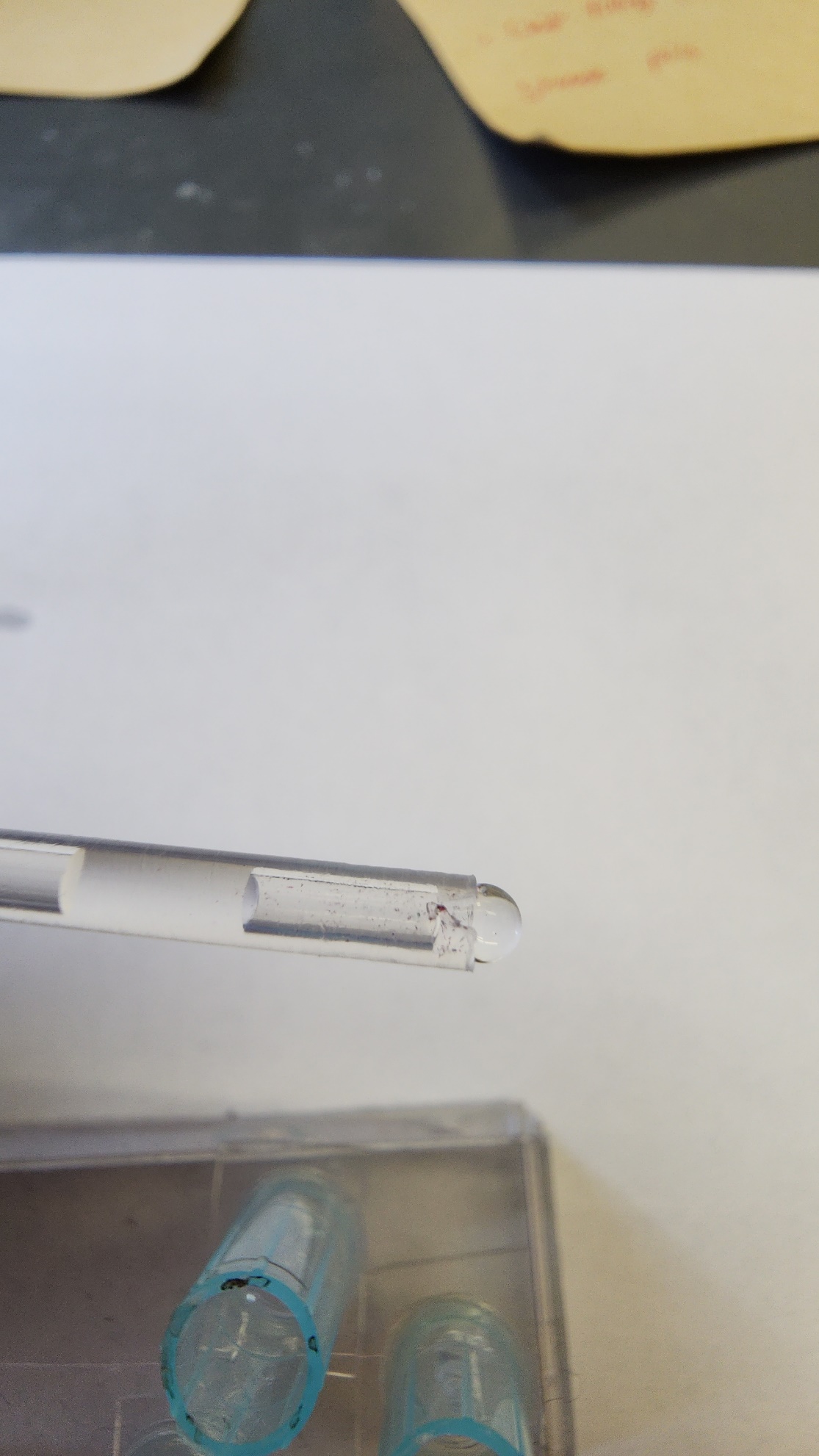


**Figure S9:** Image depicting injection/flushing of chip with vinegar/NH_4_OH

**Figure S8:** Image depicting notched end of filler syringe. When inserted in the port the, liquid enters the channel and is not blocked by the tubing.

**Cleanup:**

1. Cleanup is important to reuse the microfluidics chips

2. Chips should be well-flushed, and the channel checked under magnification for any particulate

3. Chips may be cleaned by placing them in a dilute soap bath in an ultrasonic cleaner

4. Fill chip channels with filtered vinegar

5. Store chips in refrigerator (if possible) inside a clean and sealed container (Figure S10)


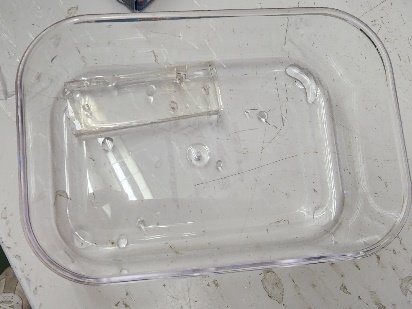


**Figure S10:** Image depicting chip stored in sealed container with channels containing filtered vinegar

| **Table S1.** Supplies for the Mini-E Experiments | | | | |
| --- | --- | --- | --- | --- |
|  | Total Cost | Quantity | Amount for 1 Expt | Unit Cost (1 expt) |
| Distilled Vinegar | $2.08 | 1.89L | 0.005L | $0.006 |
| Ammonium Hydroxide | $12.10 | 1L | 0.005L | $0.0005 |
| Allura Red AC | $67.60 | 100g |  | $0.0054 |
| Brilliant Blue FCF | $78.90 | 100g |  | $0.0019 |
| McCormick Red Food Coloring | $12.86 | 32 oz |  | $0.0032 |
| McCormick Blue Food Coloring | $6.99 | 16 oz |  | $0.0008 |
| Berkley Vanish 2Lb 0.15mm fishing line | $7.99 | 110 yds | 0.087 yds | $0.006 |
| **Total consumables** |  |  |  | **$0.024** |
|  |  |  |  |  |
| 48V DC Inverter | $10.83 | 1 | 2 | $21.66 |
| 48V DC Inverter AC Clip | $0.74 | 1 | 2 | $1.48 |
| 500 kΩ Resistor | $1.26 | 1 | 1 | $1.26 |
| Earring Hook (Electrode) | $6.42 | 8 | 2 | $1.61 |
| Multimeter | $4.99 | 1 | 1 | $4.99 |
| PDMS (Chip) | $879.00 | 3.9 kg | 19g | $4.27 |
| VHB Tape | $35.49 | 5 yds | 1 cm | $0.08 |
| 1/8”x1/8” Cylinder Magnet | $11.99 | 50 | 8 | $1.50 |
| **Total Re-useable** |  |  |  | **$36.88** |

**Consumables:**

**Distilled Vinegar**, $2.08 for 1.89L, Great Value, Walmart

<https://www.walmart.com/ip/Great-Value-Distilled-White-Vinegar-64-fl-oz/10450989?wmlspartner=wlpa&selectedSellerId=0&wl13=3215&adid=2222222227710450989_117755028669_12420145346&wmlspartner=wmtlabs&wl0=&wl1=g&wl2=c&wl3=501107745824&wl4=pla-294505072980&wl5=1028383&wl6=&wl7=&wl8=&wl9=pla&wl10=8175035&wl11=local&wl12=10450989&wl13=3215&veh=sem_LIA&gclid=Cj0KCQjw1vSZBhDuARIsAKZlijRDmTzOnvkYcQjKgnWX6x9giV8Pet6KIViSXEC9DaBwDJ6YM_EWmu4aAm9oEALw_wcB&gclsrc=aw.ds>

**Ammonium Hydroxide 1M**, $12.10 for 1L, Carolina Biological Supply, Carolina Biological Supply

<https://www.carolina.com/catalog/detail.jsp?prodId=844033&gclid=Cj0KCQjw1vSZBhDuARIsAKZlijQf8n8g8U_DmEslYjfjaXS8I4f-fp3Wo-HnfgxqpaSipEbUNt0yccsaAu07EALw_wcB>

**Allura Red AC**, $67.60 for 100g, Sigma-Aldrich, Millipore Sigma

<https://www.sigmaaldrich.com/US/en/product/sial/458848>

**Brilliant Blue FCF,** $78.90 for 100g, Supelco, Millipore Sigma

<https://www.sigmaaldrich.com/US/en/product/sial/80717>

**McCormick Red Food Coloring**, $12.86 for 32 oz, McCormick Culinary, Amazon

<https://www.amazon.com/McCormick-Food-Coloring-Red-32-Ounce/dp/B008OGD3U6?source=ps-sl-shoppingads-lpcontext&ref_=fplfs&psc=1&smid=ATVPDKIKX0DER>

**McCormick Blue Food Coloring**, $6.99 for 16 oz, McCormick Culinary, Amazon

<https://www.amazon.com/McCormick-Culinary-Blue-Food-Color/dp/B008OGD2RK/ref=pd_lpo_3?pd_rd_i=B008OGD2RK&psc=1>

**Berkley Vanish 2Lb 0.15mm Fishing Line,** $7.99 for 110 yards. https://www.dickssportinggoods.com/p/berkley-vanish-fluorocarbon-fishing-line-15bkyuvnsh25010clfli/15bkyuvnsh25010clfli?sku=10441298

**Reusable Components:**

**48V DC Inverter,** $10.83 for 1, Phihong USA, Digi-Key Electronics (PN: 993-1364-ND),

<https://www.digikey.com/en/products/detail/phihong-usa/PSAC12R-480/5418516?s=N4IgTCBcDaIJxwMwFoCMiBsAWZA5AIiALoC%2BQA48V>

**48V DC Inverter**, $10.83 for 1, Phihong USA, Digi-Key Electronics (PN: 993-1364-ND),

<https://www.digikey.com/en/products/detail/phihong-usa/PSAC12R-480/5418516?s=N4IgTCBcDaIJxwMwFoCMiBsAWZA5AIiALoC%2BQA>

**500 kΩ Resistor**, $1.26 for 1, Vishay Dale, Digi-Key Electronics (PN: 541-CMF65500K00FKEBCT-ND),

<https://www.digikey.com/en/products/detail/vishay-dale/CMF65500K00FKEB/3615118>

**Earring Hook (Electrode)**, $6.42 for 8, Cousin, Amazon.com (PN: B00872AAXK),

<https://www.amazon.com/Cousin-Jewelry-Basics-Platinum-8-Piece/dp/B00872AAXK/ref=asc_df_B00872AAXK/?tag=hyprod-20&linkCode=df0&hvadid=167125073467&hvpos=&hvnetw=g&hvrand=5911877154895872473&hvpone=&hvptwo=&hvqmt=&hvdev=c&hvdvcmdl=&hvlocint=&hvlocphy=9009439&hvtargid=pla-306014279509&psc=1&region_id=674469>

**Multimeter**, $4.99 for 1^*^, Harbor Freight Tools, Harbor Freight (PN:63759),

<https://www.harborfreight.com/7-function-digital-multimeter-63759.html?_br_psugg_q=multimeter>

**PDMS (Chip)**, $879 for 3.9 kg, DOW Corning, Amazon.com,

<https://www.amazon.com/Dow-Corning-Sylgard-184-Encapsulation/dp/B075DTMRSC/ref=asc_df_B075DTMRSC/?tag=hyprod-20&linkCode=df0&hvadid=312191701154&hvpos=&hvnetw=g&hvrand=14283520515541090016&hvpone=&hvptwo=&hvqmt=&hvdev=c&hvdvcmdl=&hvlocint=&hvlocphy=1028383&hvtargid=pla-583426527734&psc=1>

**VHB Tape**, $35.49 for 5 yards, 3M, Grainger (PN: WWG15C294)

<https://www.grainger.com/product/3M-Double-Sided-Foam-Tape-Transparent-15C294?searchQuery=WWG15C294&searchBar=true&tier=Tier+6>

**1/8”x1/8” Cylinder Magnet**, $11.99 for 50, Apex Magnets, Apex Magnets

<https://www.apexmagnets.com/magnets/1-8-x-1-8-cylinders-neodymium-magnet>
