# Supplementary material for: A Cost-Effective Microfluidic Device to Teach the Principles of Electrophoresis and Electroosmosis
Source: J Chem Educ. 2023 Jun 20;100(7):2782–8. doi: 10.1021/acs.jchemed.2c01028 (PMC10339723; doi:10.1021/acs.jchemed.2c01028)
Supplement: Supplementary file 7 — ed2c01028_si_007.pdf [file ed2c01028_si_007.pdf]

A Cost-Effective Microfluidic Device to Teach the Principles of Electrophoresis and Electroosmosis

Tyler A. Shaffer[1], Carlos U. Herrada[2], Avery M. Walker,[1] Laura D. Casto-Boggess[1], Lisa A. Holland[1]*, Timothy R. Johnson[1], Megan E. Jones,[1] Yousef S. Elshamy[1]

[1]C. Eugene Bennett Department of Chemistry, West Virginia University, Morgantown, WV 26505, United States of America

[2]Department of Chemistry, St. Norbert College, De Pere, WI 54115, United States of America

*Corresponding Author, Lisa.Holland@mail.wvu.edu

ABSTRACT

This material includes the Upper Division Student Notes.

TABLE OF CONTENTS

Information	Page
**Upper Division Student Handout**	
Part 1: Acetic acid experimental protocol	S-1
Experimental goal	S-1
Background	S-1
Procedures	S-3
Part 2: Ammonium hydroxide experimental protocol	S-6
Procedure	S-6
Reflection	S-8

Mini-E Lab: Acetic Acid Experimental Protocol

Experimental Goals. Capillary electrophoresis is an important analytical separation method for biomolecules. Before learning about capillary electrophoresis, it can be helpful to learn about the basic mechanism of electrophoretic transport. The purpose of this lab is to teach fundamental principles of electrophoresis as a means to better train students in the method of capillary electrophoresis. When a student is introduced to a capillary electrophoresis instrument in a teaching lab the time spent on the instrument will be more effective if the fundamental principles are understood.

Background. Information about capillary electrophoresis instruments and experiments is available on the Analytical Sciences Digital Library[1]. We have included instructions to build and operate capillary electrophoresis instrumentation on that website. The background on fundamental principles from that experiment is adapted in this section.

Electrophoretic transport is based on the charge attraction or repulsion of the analyte to a cathode (or anode). Cations move toward the cathode. Anions move toward the anode. The movement of these ions is affected by frictional drag. This means that Electrophoretic transport is related to the charge-to-size ratio of the analyte. Electrophoretic velocity (ν_{eph}) is the product of electrophoretic mobility (μ_{eph}), applied voltage (V), and capillary length (L).

Electroosmotic flow, depicted in Figure S1, occurs as a consequence of the application of a voltage in the presence of a surface charge in the channel. Under acidic conditions the surface is not charged (Figure S2A). Under neutral and basic conditions (Figure S2B), the PDMS has a negative charge on the surface of the PDMS channels. A negatively charged surface attracts, positive counterions, forming the Stern (static/fixed) layer (Figure S1).[2,3] These counterion originate from the background electrolyte, such as the ammonium hydroxide that is used in part 2 of this laboratory experiment. Excess solvated counterions are attracted to the PDMS surface and move towards the cathode in the diffuse layer (Figure S1).[2] This creates a drop in potential across this double layer which is proportional to the ion movement in the diffuse layer.[2,3] This movement initiates a bulk flow in the system toward the cathode.

Figure S1. Conceptual depiction of electroosmotic flow (EOF) with the anode (A) and cathode (C) indicated. The fixed layer, diffuse layer, and bulk layer are shown.

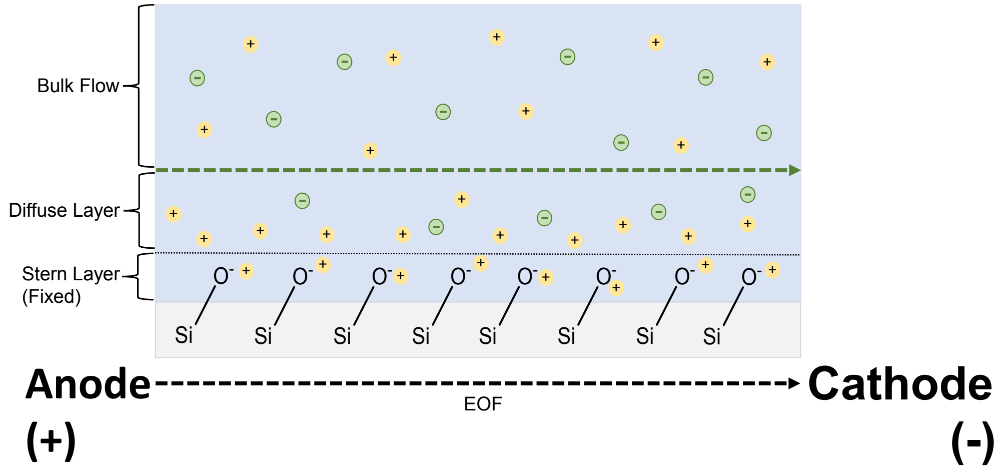

Figure S2. Panels and discussion of suppressed (A) and active (B) electroosmotic flow (EOF).

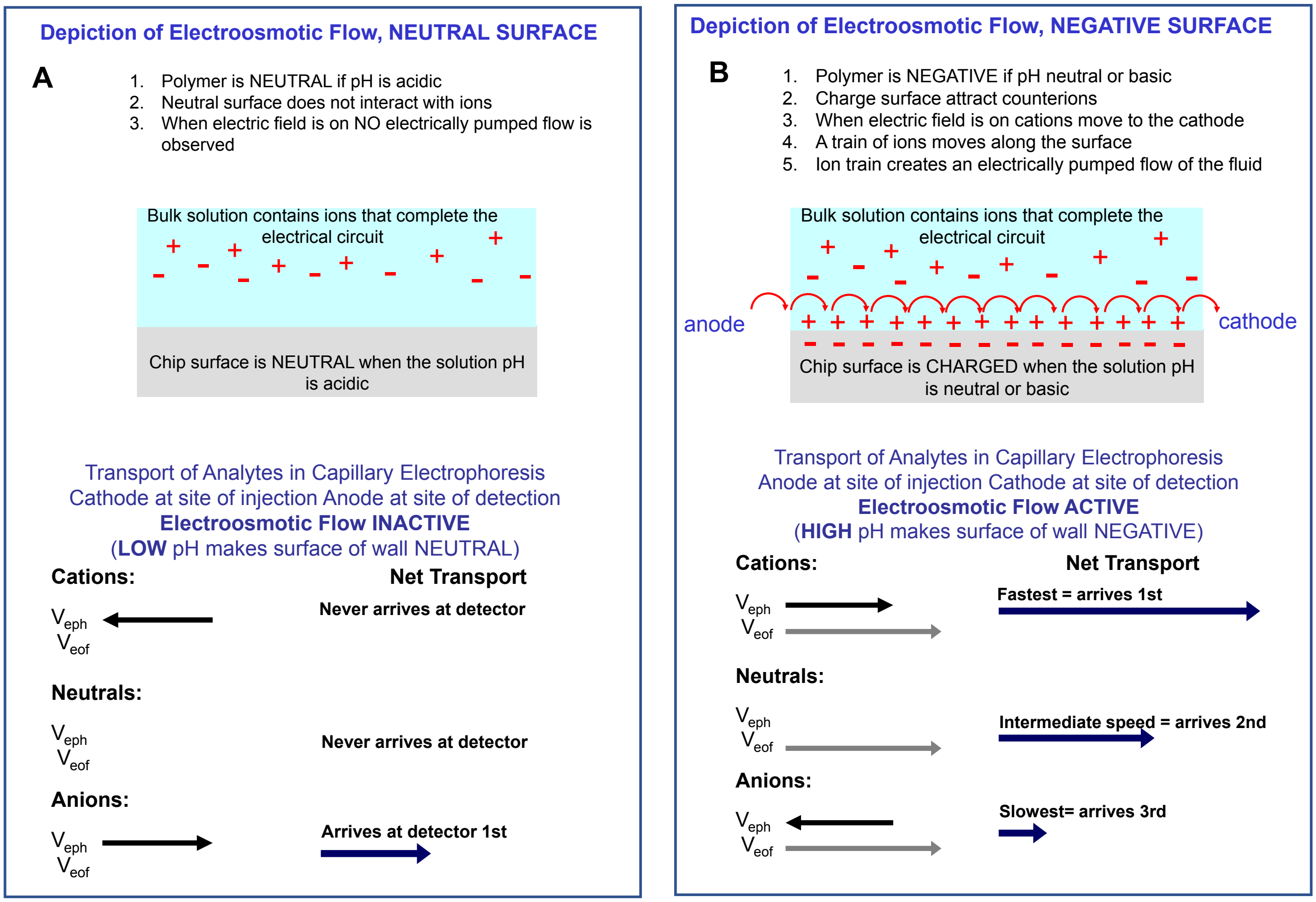

This experiment involves the use of a separation channel cast in a polymer (polydimethylsiloxane). The separation buffer is food grade vinegar, which is 0.8 M acetic acid. The analytes are two food grade dyes (Allura Red AC and Brilliant Blue FCF) shown in Figure S3.

Figure S3. Dye structures relevant to the experiments.

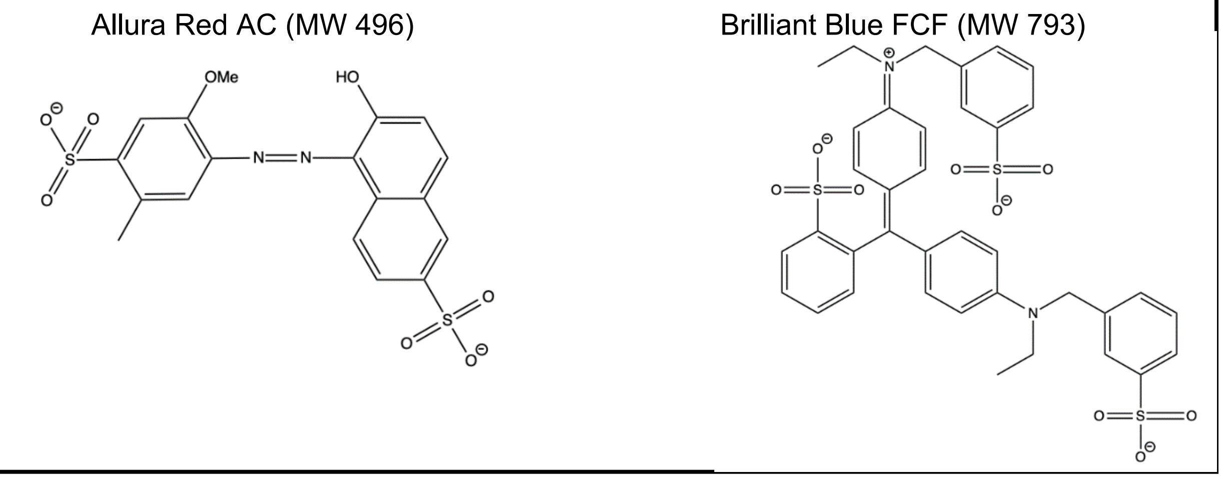

[1]C. M. White, K. M. Hanson and L. A. Holland, "MicroSeparations Distance CE: Capillary electrophoresis distance learning program: guided discovery on the principles, assembly, operation and application of a custom built capillary electrophoresis system" Analytical Sciences Digital Library: http://www.asdlib.org/ 2005 Vol. ASDL Entry 10031, available at https://collection.asdlib.org/micro-separations-distance-ce/

[2]S. Ghosal, "Fluid mechanics of electroosmotic flow and its effect on band broadening in capillary electrophoresis" Electrophoresis, 2004. 25(2): pp. 214-228.

[3]A. Alizadeh, W.-L. Hsu, M. Wang, and H. Daiguji, Electroosmotic flow: From microfluidics to nanofluidics. Electrophoresis, 2021. 42(7-8): p. 834-868.

Experimental Protocol - Vinegar (Reverse Polarity)

Students will be provided with a mini-E device. In this experiment the instructor has filled the channels with vinegar (i.e. acetic acid) prior to the student using the device. Place the device on a white background, such as a piece of paper, so that the color in the channel is more easily seen.

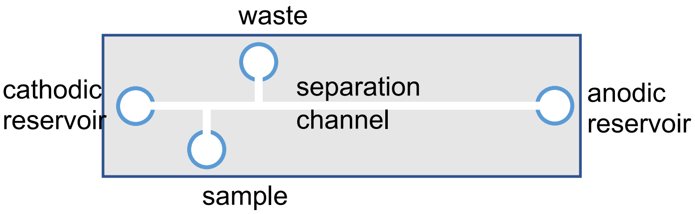

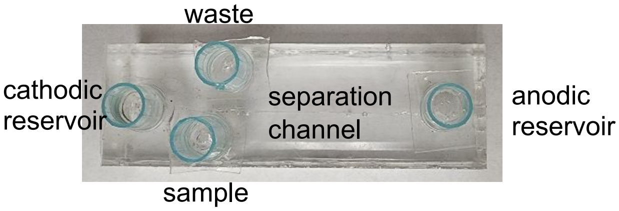

1. All wells are filled to 300 µL. Upon receiving the mini-E device check the connections between the channels by placing the electrodes in any two wells. If the channels are connected, the voltmeter will begin to measure a voltage of approximately 1.0 V or greater. **Remove electrodes when finished** and **turn power strip off**.

** When inserting the electrodes into the wells, insert them into the deepest portion of the well and make sure both the power strip and multimeter are turned on*

2a. Using a pipet, **remove 300 µL** of liquid in the of the sample well reservoir above the PDMS chip.

2b. Using a pipet, **Remove 200 µL** from the waste well.

**Be sure not to remove liquid from the lower portion of the well as this may introduce air bubbles into channels. Note the pipet is filled with blue dye in the picture below for visualization.*

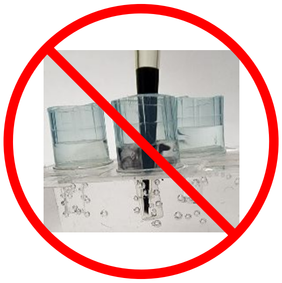

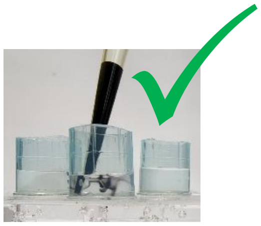

**Step 1** Well Volumes	
reservoir	volume (µL)
cathode	300 vinegar
anode	300 vinegar
sample	0
waste	300 vinegar

**Step 2** Well Volumes	
reservoir	volume (µL)
cathode	300 vinegar
anode	300 vinegar
sample	0
waste	100 vinegar

2c. Then place **300 µL of the purple acetic acid** solution into the sample well using a pipet. You should see siphoning of the dye from the sample to the waste well as in the picture below. Allow the purple solution to siphon until purple is seen in the waste well (up to 5 mins). **This simulates the process of a pressure injection in a standard capillary electrophoresis instrument but without switchable vials on the instrument autosampler.**

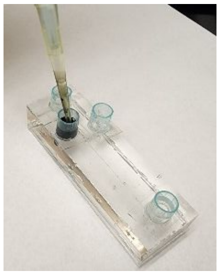

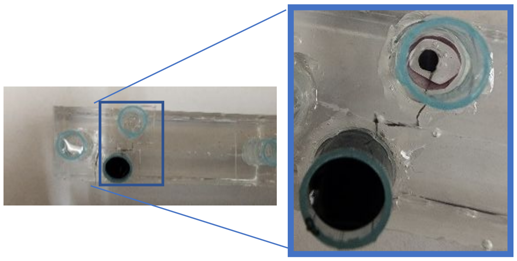

**Step 3** Well Volumes	
reservoir	volume (µL)
cathode	300 vinegar
anode	300 vinegar
sample	300 dye
waste	100 vinegar

3. Place the **cathode** into the **cathodic reservoir**. Place the **anode** into the **anodic reservoir** as shown below.

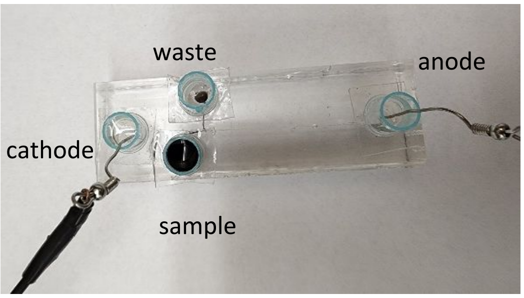

4. When the sample introduction channel is filled with color, switch on the electrophoresis **at the power strip**. Then, using a pipet, **Add 200 µL** of vinegar (**without dye**) to the waste well. The purple sample will move past the injection region and enter the main separation channel via electrophoresis.

**Step 4** Well Volumes	
reservoir	volume (µL)
cathode	300 vinegar
anode	300 vinegar
sample	300 dye
waste	300 vinegar

5. As the purple band moves along the separation channel, it will separate into blue and red colors. *Once the color separation can be seen, capture the separation with the camera on your mobile phone.*

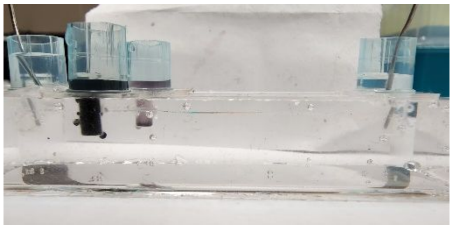

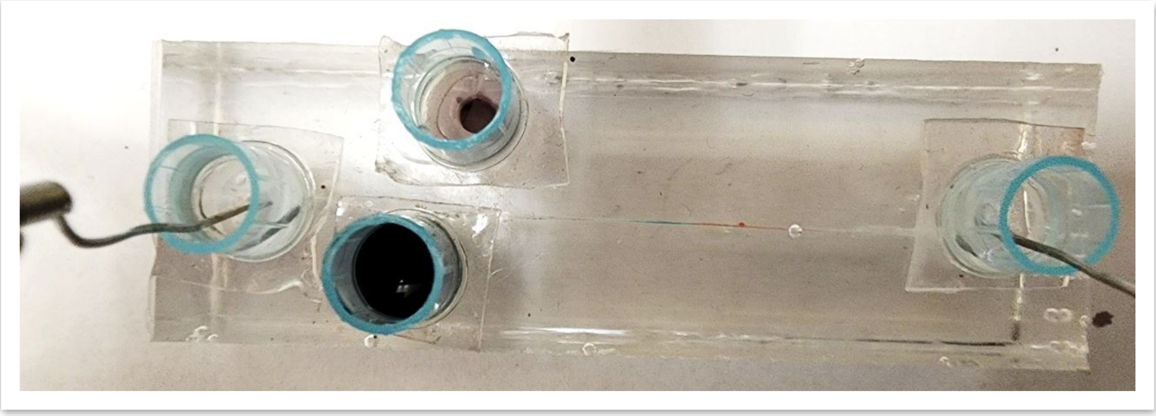

Mini-E Lab: Ammonium Hydroxide Experimental Protocol
Students will be provided with a mini-E device. In this experiment the instructor has filled the channels with 0.1N ammonium hydroxide and performed the dye sample injection prior to the student using the device. Place the device on a white background, such as a piece of paper, so that the color in the channel is more easily seen.

*** Please pay attention to the diagrams below, this portion is run in normal polarity so the anode and cathode wells are switched**

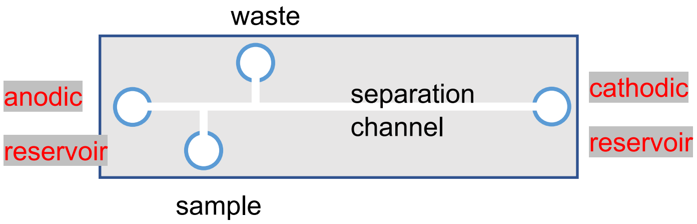

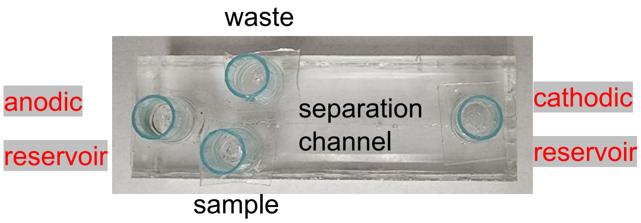

1. All wells are filled to 300 μL with ammonium hydroxide. Upon receiving the mini-E device check the connections between the channels by placing the electrodes in any two wells. If the channels are connected, the voltmeter will begin to measure a voltage of approximately 0.45 V or greater. **Remove electrodes when finished** and **turn power strip off**.

** When inserting the electrodes into the wells, insert them into the deepest portion of the well and make sure <u>both</u> the power strip and multimeter are turned on*

2. Repeat sample introduction as outlined in steps 2a,b,c for the separation in vinegar. The dye mixture must be dissolved in the ammonium hydroxide solution.

3. Place the **cathode** into the **cathodic reservoir**. Place the **anode** into the **anodic reservoir** as shown below.

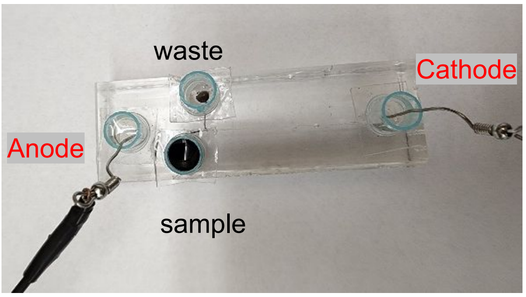

3. Switch on the electrophoresis **at the power strip**. Then, using a pipet, **Add 200 μL** of AmOH (**without dye**) to the waste well. The purple sample will move past the injection region and enter the main separation channel via electrophoresis.

4. As the purple band moves along the separation channel, it will separate into blue and red colors. *Once the color separation can be seen, capture the separation with the camera on your mobile phone.*

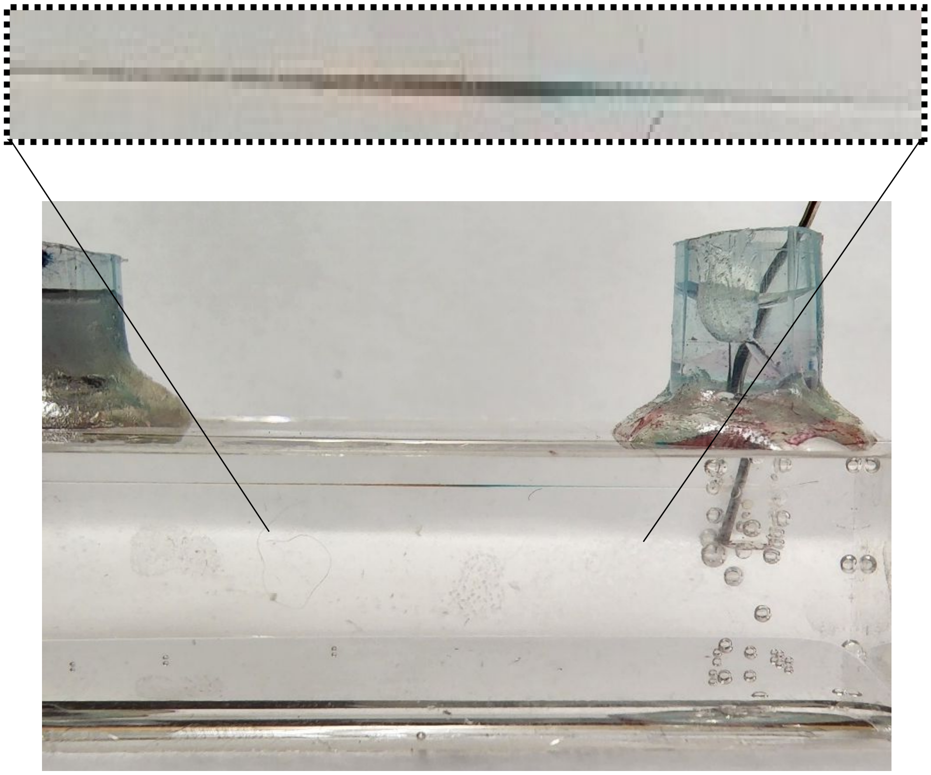

5. Using the space below, draw the vectors you observed in the experiment

Electrophoretic Mobility is a function of
Charge-to-Size Ratio
Draw the electrophoretic V_{eph} vectors below

Anion1 = brilliant blue (MW 793 net charge -2) :

V_{eph}

Anion2 = allura red (MW 496 net charge -2)

V_{eph}

Transport of Analytes in Capillary Electrophoresis
Cathode at site of injection Anode at site of detection
Electroosmotic Flow INACTIVE
(**LOW** pH makes surface of wall NEUTRAL)

Anion1 = brilliant blue:

V_{eph}
V_{eof}

Anion2 = allura red

V_{eph}
V_{eof}

Net Transport

Arrives at detector first or second?

Transport of Analytes in Capillary Electrophoresis
Anode at site of injection Cathode at site of detection
Electroosmotic Flow ACTIVE
(**HIGH** pH makes surface of wall NEGATIVE)
Draw the electrophoretic V_{eph} vectors, the electroosmotic vectors V_{eof} , and the net transport vectors below

Anion1 = brilliant blue:

V_{eph}
V_{eof}

Anion2 = allura red

V_{eph}
V_{eof}

Net Transport

Arrives at detector first or second?

6. Using the photographs taken for the acetic acid and ammonium hydroxide separations, state the order of migration. Explain why the order of migration is different for these separations.
