# Supplementary material for: A Cost-Effective Microfluidic Device to Teach the Principles of Electrophoresis and Electroosmosis
Source: J Chem Educ. 2023 Jun 20;100(7):2782–8. doi: 10.1021/acs.jchemed.2c01028 (PMC10339723; doi:10.1021/acs.jchemed.2c01028)
Supplement: Supplementary file 10 — ed2c01028_si_010.docx [file ed2c01028_si_010.docx]

**A Cost-Effective Microfluidic Device to Teach the Principles of Electrophoresis and Electroosmosis**

Tyler A. Shaffer^1^, Carlos U. Herrada^2^, Avery M. Walker,^1^ Laura D. Casto-Boggess^1^, Lisa A. Holland^1^*, Timothy R. Johnson^1^, Megan E. Jones,^1^ Yousef S. Elshamy^1^

^1^C. Eugene Bennett Department of Chemistry, West Virginia University, Morgantown, WV 26505, United States of America

^2^Department of Chemistry, St. Norbert College, De Pere, WI 54115, United States of America

*Corresponding Author, Lisa.Holland@mail.wvu.edu

**ABSTRACT**

This material includes the student handout.

**TABLE OF CONTENTS**

**Information Page**

**Introductory Student Handout**

Part 1: Acetic acid experimental protocol S-1

Experimental goal S-1

Background S-1

Procedures S-3

Part 2: Ammonium hydroxide experimental protocol S-4

Procedure S-4

Reflection S-6

**Mini-E Lab:** **Acetic Acid Experimental Protocol**

**Experimental Goals.** Capillary electrophoresis is an important analytical separation method for biomolecules. Before learning about capillary electrophoresis, it can be helpful to learn about the basic mechanism of electrophoretic transport. The purpose of this lab is to teach fundamental principles of electrophoresis as a means to better train students in the method of capillary electrophoresis. When a student is introduced to a capillary electrophoresis instrument in a teaching lab the time spent on the instrument will be more effective if the fundamental principles are understood.

**Background.** Information about capillary electrophoresis instruments and experiments is available on the Analytical Sciences Digital Library^1^. We have included instructions to build and operate capillary electrophoresis instrumentation on that website. The background on fundamental principles from that experiment is adapted in this section.

Electrophoretic transport is based on the charge attraction or repulsion of the analyte to a cathode (or anode). Cations move toward the cathode. Anions move toward the anode. The movement of these ions is affected by frictional drag. This means that Electrophoretic transport is related to the charge-to-size ratio of the analyte. Electrophoretic velocity (*ν*_eph_) is the product of electrophoretic mobility (μ_eph_), applied voltage (V), and capillary length (L).

This experiment involves the use of a separation channel cast in a polymer (polydimethylsiloxane). The solution used to fill the channel for the separation for experiments performed under acidic conditions is 0.2 micron filtered food grade vinegar, which is 0.8 M acetic acid. The solution used to fill the channel for the separation for experiments performed under basic conditions is 0.1 M ammonium hydroxide. The analytes are two different food grade dyes (Allura Red AC and Brilliant Blue FCF) shown below. The dye standards should be prepared in the background electrolyte (i.e. 0.8M acetic acid OR 0.1 M ammonium hydroxide) to a concentration of 60 mM Allura Red AC and 40 mM Brilliant Blue FCF.

Allura Red AC (MW 496) Brilliant Blue FCF (MW 793)


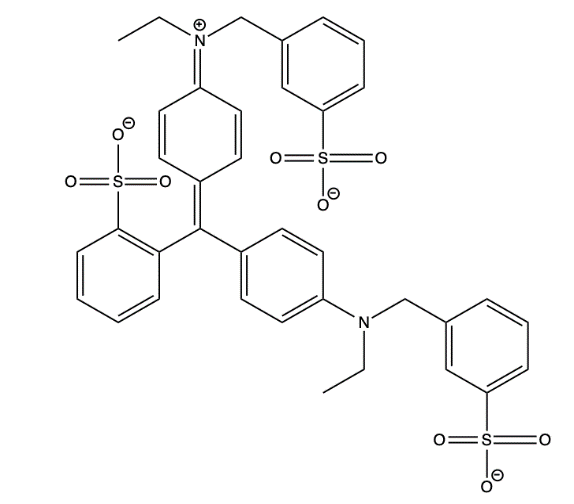


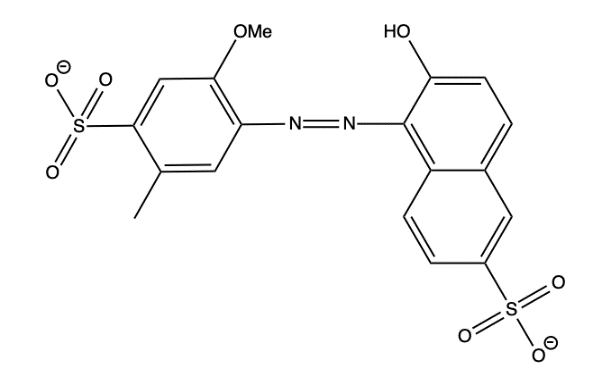


^1^C. M. White, K. M. Hanson and L. A. Holland, “MicroSeparations Distance CE: Capillary electrophoresis distance learning program: guided discovery on the principles, assembly, operation and application of a custom built capillary electrophoresis system”

Analytical Sciences Digital Library: <http://www.asdlib.org/> 2005 Vol. ASDL Entry 10031, available at <https://collection.asdlib.org/micro-separations-distance-ce/>

**Experimental Protocol** - Vinegar (Reverse Polarity)

Students will be provided with a mini-E device. In this experiment the instructor has readied the device with acetic acid and dye solution prior to the student using the device. Place the device on a white background, such as a piece of paper, so that the color in the channel is more easily seen.


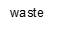
**
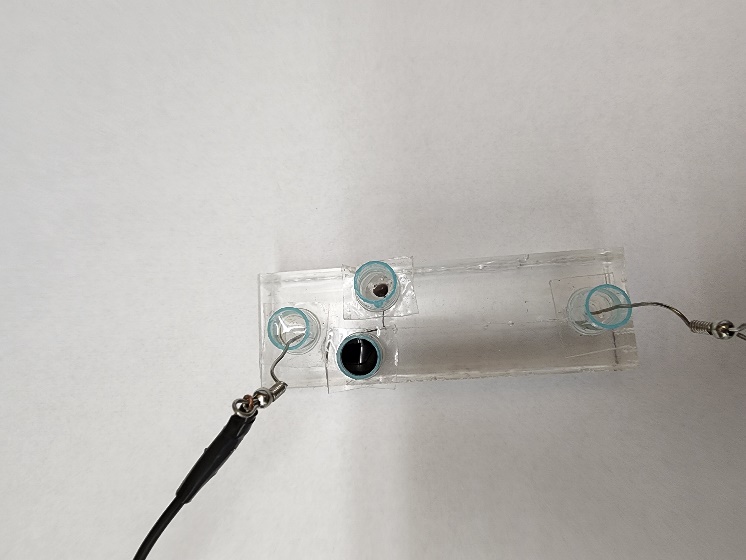
1.** Place the **cathode** into the **cathodic reservoir**. Place the **anode** into the **anodic reservoir** as shown below.

anodic

reservoir

separation channel

cathodic

reservoir

sample

sample

waste

anode

cathode

* When inserting the electrodes into the wells, insert them into the deepest portion of the well and make sure both the power strip and multimeter are turned on. When the power is on and current is flowing across the channels between the ports in which the electrodes are inserted, then the multimeter will read a voltage. If no voltage is measured, it may be necessary to flush or refill the channel.

**2.** When the sample introduction channel is filled with color, switch on the electrophoresis **at the power strip**. Then, using a pipet, **Your instructor will add 200 μL** of vinegar (**without dye**) to the waste well. The purple sample will move past the injection region and enter the main separation channel via electrophoresis.

**3.** As the purple band moves along the separation channel, it will separate into blue and red colors. *Once the color separation can be seen, capture the separation with the camera on your mobile phone.*

**
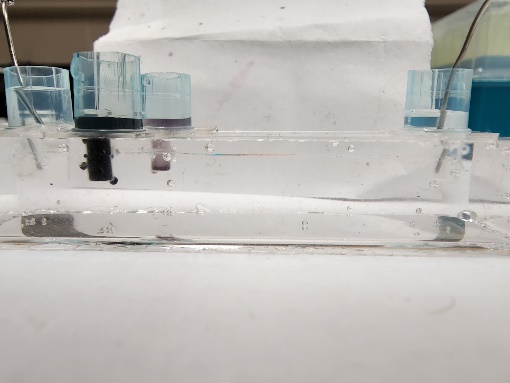
**

**
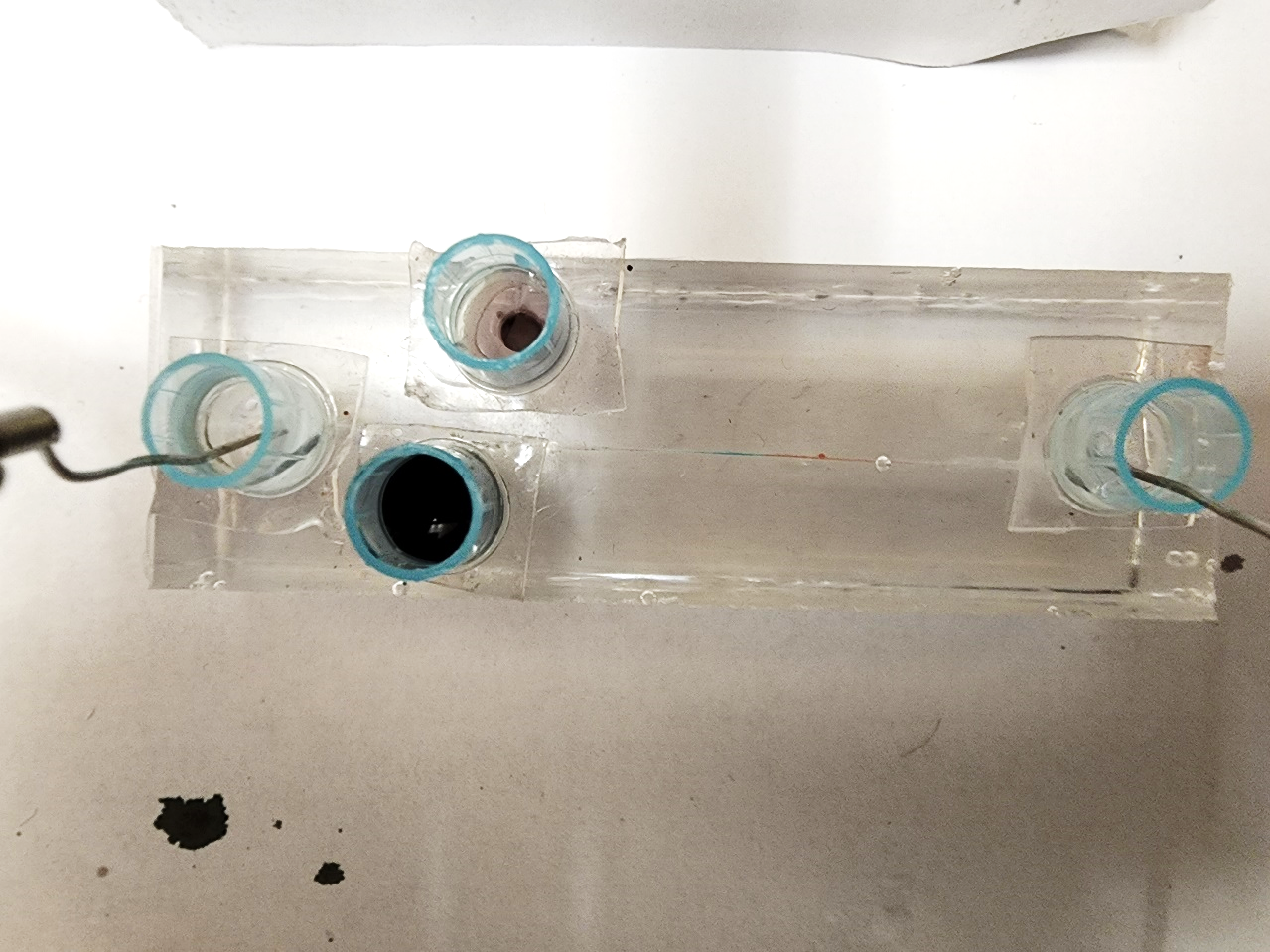
**

**Experimental Protocol** – Ammonium Hydroxide (Normal Polarity)

Students will be provided with a mini-E device. In this experiment the instructor has readied the device with 0.1N ammonium hydroxide (AmOH) and dye solution prior to the student using the device. Place the device on a white background, such as a piece of paper, so that the color in the channel is more easily seen.

*** Please pay attention to the diagrams below, this portion is run in normal polarity so the anode and cathode wells are switched**

**
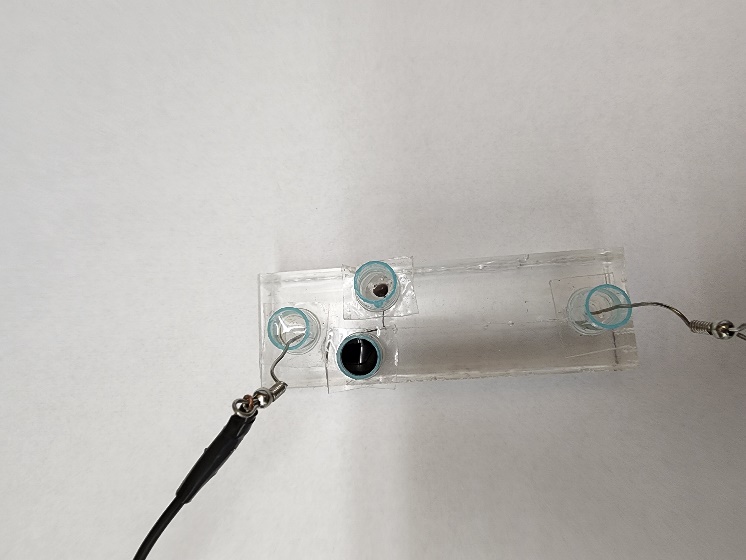
1.** Place the **cathode** into the **cathodic reservoir**. Place the **anode** into the **anodic reservoir** as shown below.

cathodic

reservoir

anodic

reservoir

waste

sample

separation channel

waste

Anode

Cathode

sample

* When inserting the electrodes into the wells, insert them into the deepest portion of the well and make sure both the power strip and multimeter are turned on. When the power is on and current is flowing across the channels between the ports in which the electrodes are inserted, then the multimeter will read a voltage. If no voltage is measured, it may be necessary to flush or refill the channel.

**2.** Switch on the electrophoresis **at the power strip**. Then, using a pipet, **Your instructor will add 200 μL** of AmOH (**without dye**) to the waste well. The purple sample will move past the injection region and enter the main separation channel via electrophoresis.

| Table of Well Volumes | |
| --- | --- |
| reservoir | volume (µL) |
| cathode | 300 AmOH |
| anode | 300 AmOH |
| sample | 300 dye |
| waste | 300 AmOH |

**
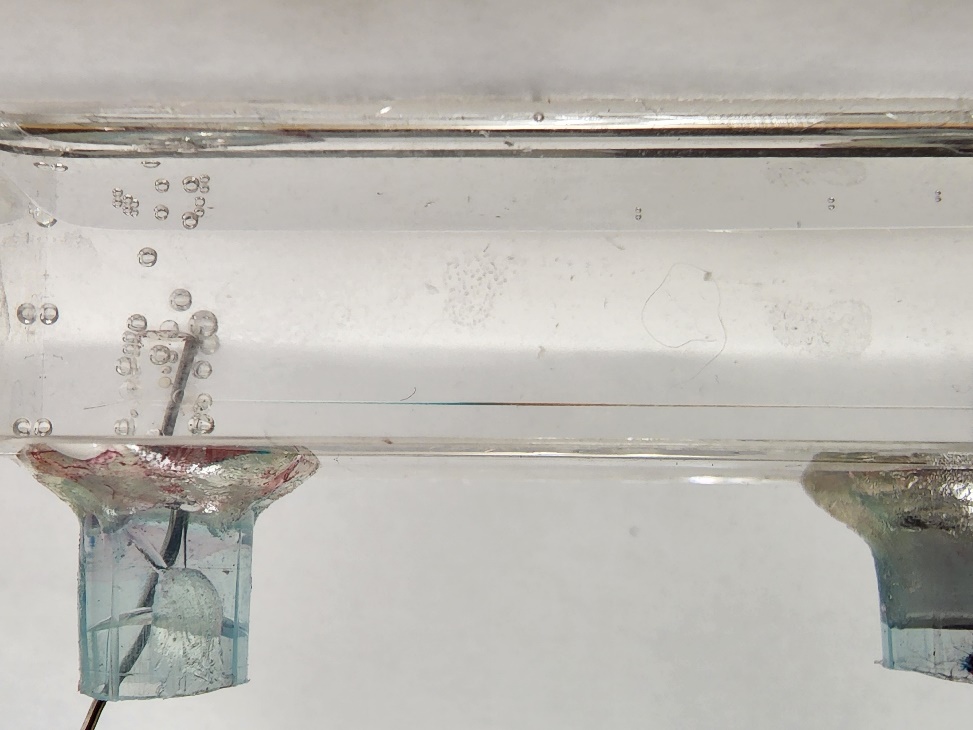
**

**3.** As the purple band moves along the separation channel, it will separate into blue and red colors. *Once the color separation can be seen, capture the separation with the camera on your mobile phone.*

**4.** Using the space below, draw the vectors you observed in the experiment

**5.** Using the photographs taken for the acetic acid and ammonium hydroxide separations, state the order of migration. Explain why the order of migration is different for these separations.
