# Supplementary material for: A Cost-Effective Microfluidic Device to Teach the Principles of Electrophoresis and Electroosmosis
Source: J Chem Educ. 2023 Jun 20;100(7):2782–8. doi: 10.1021/acs.jchemed.2c01028 (PMC10339723; doi:10.1021/acs.jchemed.2c01028)
Supplement: Supplementary file 11 — ed2c01028_si_011.pdf [file ed2c01028_si_011.pdf]

A Cost-Effective Microfluidic Device to Teach the Principles of Electrophoresis and Electroosmosis

Tyler A. Shaffer[1], Carlos U. Herrada[2], Avery M. Walker,[1] Laura D. Casto-Boggess[1], Lisa A. Holland[1]*, Timothy R. Johnson[1], Megan E. Jones,[1] Yousef S. Elshamy[1]

[1]C. Eugene Bennett Department of Chemistry, West Virginia University, Morgantown, WV 26505, United States of America
[2]Department of Chemistry, St. Norbert College, De Pere, WI 54115, United States of America

*Corresponding Author, Lisa.Holland@mail.wvu.edu

ABSTRACT

This material includes the author contributions.

Author contributions are written according to: Brand, A.; Allen, L.; Altman, M.; Hlava, M.; Scott, J., Beyond authorship: attribution, contribution, collaboration, and credit. Learned Publishing 2015, 28 (2), 151-155.

<u>Author contributions are as follows:</u>

TAS: Conceptualization-Fishing line channels and pipette tip wells electrics design, Methodology-(final casting technique, sample injection, cleanup technique, assessment), Validation (assessment), Formal Analysis-all data, Investigation- (all data, separation methods, dye concentration, assessment) Resources- electrical setups and chips, Data curation, Writing – Original Draft, Writing – Review & Editing, Visualization, Supervision

CH: Methodology-(final casting technique, sample injection), Validation (assessment), Formal Analysis- initial data, Investigation- (initial data, Dye Concentration studies, separation methods) Resources – solution prep, Visualization

AMW: Methodology (Casting), Validation (vinegar separations of dye mixtures), Investigation-band broadening and commercial dye use in current setup, fishing line casting, Visualization (Figure 4A,B,C), Writing – Review & Editing.

LDC-B: Conceptualization-microfluidic practices, final well design, Methodology-(Injection strategies, well design, VHB Tape) Formal analysis (assessment statistical testing), Investigation-final data

LAH: Conceptualization, Methodology-(sample injection, cleanup technique, assessment), Validation (assessment), Investigation-gathering all data (assessment), Writing – Original Draft, Writing – Review & Editing, Visualization, Supervision, Project Administration, Funding acquisition

TRJ: Methodology- casting basics, assessment

MEJ: Methodology (Casting), Validation (vinegar separations of dye mixtures).

YSE: Investigation (studies of separation currents), Resources- solutions.
